# The Application of LiDAR to Assessment of Rooftop Solar Photovoltaic Deployment Potential in a Municipal District Unit

**DOI:** 10.3390/s120404534

**Published:** 2012-04-10

**Authors:** Ha T. Nguyen, Joshua M. Pearce, Rob Harrap, Gerald Barber

**Affiliations:** 1 Department of Geography and Environment, Boston University, Boston, MA 02215, USA; E-Mail: nguyetha@bu.edu; 2 Department of Materials Science & Engineering and Department of Electrical & Computer Engineering, Michigan Technological University, 1400 Townsend Dr., Houghton, MI 49931, USA; 3 Department of Geological Sciences & Geological Engineering, Queen's University, Kingston, ON K7L 3N6, Canada; E-Mail: harrap@mac.com; 4 Department of Geography, Queen's University, Kingston, ON K7L 3N6, Canada; E-Mail: barberg@queensu.ca

**Keywords:** airborne laser scanning, ALS, Digital Surface Model, DSM, Light Detection and Ranging, LiDAR, roof extraction, photovoltaic

## Abstract

A methodology is provided for the application of Light Detection and Ranging (LiDAR) to automated solar photovoltaic (PV) deployment analysis on the regional scale. Challenges in urban information extraction and management for solar PV deployment assessment are determined and quantitative solutions are offered. This paper provides the following contributions: (i) a methodology that is consistent with recommendations from existing literature advocating the integration of cross-disciplinary competences in remote sensing (RS), GIS, computer vision and urban environmental studies; (ii) a robust methodology that can work with low-resolution, incomprehensive data and reconstruct vegetation and building separately, but concurrently; (iii) recommendations for future generation of software. A case study is presented as an example of the methodology. Experience from the case study such as the trade-off between time consumption and data quality are discussed to highlight a need for connectivity between demographic information, electrical engineering schemes and GIS and a typical factor of solar useful roofs extracted per method. Finally, conclusions are developed to provide a final methodology to extract the most useful information from the lowest resolution and least comprehensive data to provide solar electric assessments over large areas, which can be adapted anywhere in the world.

## 1. Introduction

Solar photovoltaic (PV) energy conversion offers a sustainable method of producing electricity to provide for contemporary society's needs [1]. The advantages of PV in producing electricity are: (i) no atmospheric emissions or radioactive waste generation during use; (ii) it acts as a distributed electrical generation source and hence reduces the dependence and pressure on the central utility lines in systems with high potential for blackouts and overloads; (iii) assistance in national energy security [2,3] and (iv) long–term economic growth improvement [4–7] for any country that aggressively develops the technology. This has led to international cooperation and technology investment over the past 25 years, which in turn has given rise to fantastic gains in solar PV cell performance and a predicted changing landscape in R&D activities for solar cell technologies [8–11]. Solar cells made from a variety of materials have demonstrated efficiencies over ten percent and are currently manufactured globally.

As the technological proficiency of the solar cell industry matured, the total shipments of solar cells increased rapidly. In the last decade (to 2010), global solar PV deployment has increased from <1 GW to >16 GW with an annual growth rate of more than 40% [12–16]. This growth rate, while impressive, must be kept in context of the global energy market. In 2000 the peak electrical generation capacity in the U.S. was 825 GigaWatts (GW = 10^9^W), while the cumulative total global installed solar PV was less than a single GW. The technical potential for solar PV within the first half of the 21st century is predicted to grow by 4 times to reach 4,195 PWh/ year by 2050 [17].

The increasing technological competitiveness of solar PV, among other kinds of renewable energies, has contributed to a “new logic of infrastructure provision” [18] and a “paradigm shift in energy policy” [19]. However, in the debates on urban and regional development and regional infrastructure policy, the integration of different disciplines (geographic information system (GIS), environmental modeling, urban planning, electrical and mechanical engineering) and their roles in the delivery of utility services still seems to be taken for granted by the public and within each single discipline and to be left to engineers, network operators and (supra-) national utility regulators. Consequently, there has been little research on the urban and regional impacts of utility restructuring and the changing environment for urban and regional governance [20,21] with a large-scale introduction of PV. To take advantage of PV technology's continued price declines, an understanding of the urban local potential (roof space and solar exposure among others) is critical for utility planning, accommodating grid capacity, deploying financing schemes and formulating future adaptive policies [22].

The paper describes a methodology that is part of the complex process of assessing solar PV potential for a region using the Renewable Energy Region (RER) of Southeastern Ontario as an example [22,23]. Specifically the methodology provides an application of Light Detection and Ranging (LiDAR) of urban terrain to automated solar PV deployments on a municipal unit, which can be scaled up first to the level of a city and then the cities within the RER region. The primary stakeholders for this research are local and regional utilities companies (e.g., Utilities Kingston), municipal government (e.g., the City Council of Kingston) and academic research on regional energy modeling (e.g., Queen's University and GEOIDE). Challenges in urban information extraction and management for solar PV deployment assessment are determined and quantified. This study provides the following contributions: (i) a methodology that integrated the cross-disciplinary competences in remote sensing (RS), GIS, computer vision and urban environmental studies; (ii) a robust methodology that can work with low-resolution, spatially and temporally inconsistent and incomprehensive data and reconstruct vegetation and buildings separately and concurrently; (iii) recommendations for future generations of software. It then presents a case study as an example of the methodology applied to realistic, complex data for Kingston, Ontario. Experience from the case study such as trade-offs between time consumption and data quality is discussed. This discussion highlights a need for connectivity between demographic information, electrical engineering schemes and geographical information systems (GIS) and a typical factor of solar PV suitable roof area that can be extracted per method. Finally conclusions are developed to provide guidelines for a final methodology with the most useful information in situations of incomprehensive GIS data to facilitate the processing of LiDAR, low budgets for both time and finance, and personnel with diverse expert in computer vision. The methodology can be adapted for use anywhere that LiDAR and urban GIS data is available.

## 2. Background

### 2.1. LiDAR and the Cityscape

The proverbial Holy Grail of the urban remote sensing research community is the ability to quickly and easily build accurate 2D and 3D representations of urban areas. One approach is to take a LiDAR point cloud and transform it into a high-resolution and accurate 2.5D model of the scanned area. Unfortunately, although the promise of 3D, urban texture reconstruction from laser scanning (and images) has been predicted for some time [24] with predictions of novel platforms and methodologies soon to come on board, an automated determination of reliable and accurate city models from Airborne Laser Scanning (ALS) data is still a challenging task, requiring a complex multi-stage workflow, and in some cases, combining both photogrammetry and ALS [25,26]. However the prospective applications of city models in urban development and management, for example renewable energy planning in urban centers [26,27], realistic visualization of the 3D urban environment [28], sustainable urban design [26], gaming, disaster management and military training [29], microclimate investigation, run-off modeling, telecommunications and noise simulation [30] are such that any step towards replacing the use of commercialized (and hence often costly) software, minimizing the degree of human interaction, and streamlining the process is highly desirable.

In order to determine PV potential for a city the ideal circumstance is having access to a 3D urban model, which requires that individual buildings are represented, next to urban vegetation, streets, and other objects of the city infrastructure such as watercourses, power supply lines, and individual objects like street signs or fountains. A Digital Surface Model (DSM) derived from point clouds acquired by LiDAR or stereophotogrammetry will indirectly represent buildings. While such models can be generated easily and even automatically, they only represent the approximate roof shapes without generalization and without distinguishing between individual buildings on the one hand and between buildings and other objects like ground and vegetation on the other hand. If building or building block outlines (e.g., from cadastral maps) are provided, models extracted from the combined LiDAR and GIS data are enhanced and surface models can be generated for individual buildings or blocks. However, these models still do not allow a distinction between individual roof faces, nor between roof and dormers or other objects, which is important in the context of solar PV installations because a whole roof PV installation is not always feasible. Furthermore, artifacts of data acquisition, e.g., caused by occluded areas, sampling distance, or remaining geo-referencing errors, are typically found in such models. Vertical walls may appear slanted or not appear at all due to the 2.5D grid representation [25,31]. After the clean-up of such errors, roof sizes will generally be smaller than their actual sizes, thereby reducing their shadowing potential. In the extreme cases, shadow effects of adjacent buildings may not be captured.

To increase the reliability of the building models as well as the range of possible applications, additional knowledge on buildings has to be incorporated into the modeling process. Typical assumptions are to define walls as being vertical and roofs as being a composite of planar faces. This leads to an idealization of the buildings. The transition zone of two neighboring roof faces, for example, becomes a straight line defined by the intersection of two roof planes. The importance of these considerations is raised when it comes to PV system design and per roof installation: this enhanced model is sufficient input for rapid roof assessment [32]. Hence a modeling method is needed for PV applications that is: (i) accurate, *i.e.*, it should produce simple polygonal models fitting the input point clouds in a precise manner; (ii) robust: regardless of the diversity and complexity of building roof shapes the method should always generate building models comprised of flat planes that are as continuously and smoothly connected and transitioned as possible even with the existence of undesired elements such as residual noise and small roof features; and (iii) complementary to the 2.5D characteristic: the method should create 2.5D polygonal models composed of detailed roofs and vertical walls connecting roof layers [33].

### 2.2. Building Detection

Reliable and accurate building generation from LiDAR data requires a number of processes beyond capture of accurate raw data. These are building detection, object segmentation, building extraction, roof shape reconstruction and modeling quality analysis. The majority of available literature: (i) concentrates on individual aspects only [25,26] and hence detaches the reader from the big picture; (ii) only uses (associated) data of certain (excellent) quality [26,34] or customized classifiers that are only professionally known [35] or not publicly available (eCognition, Feature Analyst); and (iii) has limited applications in terms of the degree of complexity that the product allows [25,36]. Although urban texture modeling is highly dynamic (due to the nature of the modeled object) and complicated (due to the required precision and interdisciplinary in the number of involved expertise and the inherent interaction between humans and the urban environment), urban texture influences energy consumption and production patterns [37–39] and therefore requires modeling skills and precision in order to produce informative output. How much detail can be learned about the output depends on how detailed the reconstructed urban scene is but also the budget for time, technology and personnel. The methodology for building detection subsequently derived and described here was designed to compromise between cost savings and the smoothest and most effectively established learning curve possible for an audience assumed to have no previous training in computer programming, remote sensing or digital image processing. An understanding of the complexity of GIS data integration in energy modeling means an improved inter-disciplinary appreciation for the value of a streamlined and comprehensive data system. Further, the ability to carry out part of the procedure will help facilitate the penetration of solar PV into the current electricity grid.

In the absence of cadastral data, there are several effective methods for building detection that work well on larger buildings although smaller buildings are often missed [40–43]. Building detection can be carried out solely on the LiDAR data [25] or in hybrid with other data such as aerial photos and existing building outlines [44]. Building outlines are the intersection of the buildings with its surroundings, in general the terrain. Some authors have suggested the use of polygons originating from official cadastral databases [45,46], which usually are the only available data sources. Compared to official cadastral data, which are often generated from a combination of photographic restitution and hand drawn processing by a local government employees, the outlines that are used for the approach used here have two advantages: firstly, they represent the real shape and size of the roof, and not the dimension of the basement. And secondly, applying the same data as used for the evaluation ensures the comparability of the results during the process of the evaluation [44]. Kaartinen *et al.* [47] compare the performance of photogrammetric, laser scanning based and hybrid methods in building extraction within a European Spatial Data Research (EuroSDR) test. They concluded that laser scanning is more suitable than traditional photogrammetry for deriving building heights, extracting planar roof faces and ridges of the roofs. However, photogrammetry and aerial images lead to better results in building outline and length determination.

On a more sophisticated mathematical level, Ahmadi *et al.* [48] corrected satellite images and then applied active contour models developed by Kass *et al.* [49] to achieve a level of 96% correct extraction out of 341 buildings. When building outlines are available, such as in the case given below, the LIDAR data is masked by the outlines of the buildings in order to obtain—by way of exclusion—those points that carry information about the elevation of the buildings' roofs. This necessarily assumes that the LiDAR and outline data are well aligned, that features do not overlie a rooftop (*i.e.*, fall within the outline without being part of the roof), and that the inherent accuracy of all data is considered.

### 2.3. Building Segmentation

Segmentation provides an excellent starting point for subsequent geospatial analyses [50,51]. The segmentation in the following context retains the same merits towards subsequent geospatial and statistical analysis but its object is LiDAR point determinations. Although Dorninger and Pfeifer [25] defined segmentation as a decomposition of a building as represented in a laser scanning point cloud into planar faces and other objects, for a raw 3D point cloud, segmentation oftentimes means decomposition of a point cloud into subclouds corresponding to individual buildings, then a subcloud into individual planes and separation of such objects as roof hanging, turrets, trees growing over the roof, *etc.* The handling of trees is a challenge on its own and will need to be treated elsewhere. But to continue with the discussion of segmentation, such wide contextual consideration is particularly true in downtown and near downtown areas, where even cadastral derived building roofprints tend to group houses together. The segmentation is also advantageous in that it breaks down the work in cachet-manageable chunks *i.e.*, less computationally overloading, allows for noise and artifact removal at the unit level and minimizes the task of roof fitting later by omitting any obvious anomalies.

Both block to building and building to plane segmentations can be realized via iterative evaluation of the one-dimensional marginal distributions in elevation of points making up the feature space. The latter requires the definition of a homogeneity (coplanarity) criterion according to which similar items (e.g., points) are grouped [25]. As homogeneity criterion, approximate height similarity or/and approximate normal vector similarity are commonly used.

Segmentation often does not include a distinction between flat and complex roofs. Alexander *et al.* [30] used derived information (slope, aspect, elevation) from the LiDAR data via Triangulated Irregular Network generation (Delaunay triangulation) and from building polygons via geometric conversion (polygon, multi-line, and line) to separate flat roof and pitched roof buildings. The attempt suffers from inherent elevation noise of the LiDAR data and the assumption that derived aspects are reliable (while it is never the case) and hence the result was only suitable for visualization purposes. It should be noted, the segregation of multiple roof planes using segmentation [52] and robust splitting [53] have also been shown to be effective methods. Whether the segmentation is model driven [54] or data driven [55], a subsequent refinement of individual point clouds via local histogram analysis and thresholding always follows.

A systematic comparison of the performance of methods proposed for roof face segmentation, in particular between iterative and direct approaches, is given in Nyaruhuma [56]. It was concluded that iterative methods (least squares, Hough transformation, Triangulated Irregular Network [TIN]) have generally shown encouraging results but they are mainly affected by the presence of outliers or noise in the point clouds and may be computationally expensive due to processing through iterations that increase with increases in point spacing and data size. For most simple roofs, both the Hough and TIN algorithms perform well, with the superiority yielding to the former. With increased complexity in roof structure, Hough performs better than TIN, and even more at higher tolerance level. The time taken for TIN processing increases proportionately with the increased number of points. That is because the number of triangles which are the starting segments for the algorithm, and which are joined one after the other iteratively, increases with increasing data size. Given the same large data set, TIN took an hour, as opposed to a few seconds by the Hough transformation [56]. Timing has established the superiority of Hough over TIN, which was in turn demonstrated as less preferable by Tarsha-Kurdi *et al.* [57–59].

Tarsha-Kurdi *et al.* [57–59] pointed out the implicit assumptions of the catalogue driven/ data driven building constructing models; moving on to compare the Hough transformation with Random Sample Consensus (RANSAC) and then focused on the performance and improvement of RANSAC. Although both approaches consider that a primitive building can be described as a set of parameters, the model driven methods calculate the values of the parameters before constructing the 3D model after a chosen model out of a catalog whereas the data driven ones simulate each part of the point cloud in order to obtain the nearest possible polyhedral model, and then obtaining the parameters of the fitted planes. Region growing algorithms were bypassed since they are sometimes not very transparent and not homogeneously applied. However, the 3D Hough transform can be mathematically obscure to first time users and sometimes only return the best statistically represented planes. The method only looks for the plane containing the maximum number of points, not necessarily the most fitting plane calculated according to the least squares theory nor their geometric significance with the actual building.

For this reason the modified RANSAC (mRANSAC) was designed and used here, which maintains the same principle, it searches the best plane among a 3D point cloud by randomly selecting three points, calculating the parameters of the corresponding plane and iterating through the set while minimizing the number of iterations. The number of points belonging to a calculated plane is set to at least equal to a given threshold. This threshold plays an important role in determining the efficiency and accuracy of the algorithm and is set interactively for each point subcloud.

### 2.4. Building Reconstruction

After individual cloud segments that correspond to building faces are recognized a method is needed to interpolate the heights in between the points and hence transform the working geometry from points to polygons. The methods pertaining to this purpose are called building (or object) reconstruction. Dorninger and Pfeifer [25], Tarsha-Kurdi *et al.* [57–59] among others divided building reconstruction methods into two fundamentally different approaches: model driven and data driven. In model driven methods a predefined catalog of roof forms is prescribed. The models are tested and the one with the best fit is chosen [54,60]. This is especially appropriate for low point densities. An advantage is that the final roof shape is always topologically correct. A disadvantage is, however, that complex roof shapes cannot be reconstructed, because they are not included in the catalog.

Data driven methods are appropriate for high point densities [31], whereby the roof is “reassembled” from roof parts found by segmentation algorithms. The results of the segmentation process are sets of points, each one ideally describing exactly one roof face. Some roof elements (e.g., small dormers, chimneys, *etc.*) may not be properly detected, for example only partially reconstructed or entirely missed. An important assumption here is that small windows on the roof, overhangs on the walls, HVAC equipment and antenna masts do not occupy so large a space that their omission adds significant area to the roof area free for panels, nor do they impose a significant amount of shading on future panels once installed. With such assumptions, the omission ceases to be a problem as the key is that features are reconstructed at a level of detail and accuracy appropriate for PV solar deployment studies.

The paper avoided the challenge in identifying neighboring segments and the start and end point of their intersection by way of partitioning the given ground plane and finding the most appropriate (in some cases: nearest) plane segment to each partition [61].

## 3. Methodology

As outlined above, a wide range of techniques have been used to extract building geometry, and in particular roof geometry, from LiDAR point clouds and from imagery with or without independent building outline data. Our focus is on rapid, efficient, and computationally “light” extraction of roof models to assess photovoltaic solar potential of neighborhoods. Unlike most of the methods described above, exactness is not deemed as essential as is generality to a wide variety of situations and efficiency in spite of realistically mixed data quality.

The methodology used herein is based on the following five assumptions: (i) individual building roof areas can be modeled properly by a composition of planer faces; (ii) anything below a chosen elevation cutoff is irrelevant for solar PV potential assessment; (iii) tree canopies are opaque; (iv) small windows on the roofs, overhangs on the walls, Heating, Vent and Air Conditioning facilities (HVACs) and antennas do not occupy so large a space that its omission adds significant area to the roof area free for PV panels; (v) the height of the object and subsequently the altimetry of the Digital Surface Model (DSM) is the difference between LiDAR's z values and an available DEM and (vi) there is no discrepancy in the form of urban structures between aerial photos (AP) and LiDAR (*i.e.*, these datasets were collected at or near to the same time).

**Figure 1.**
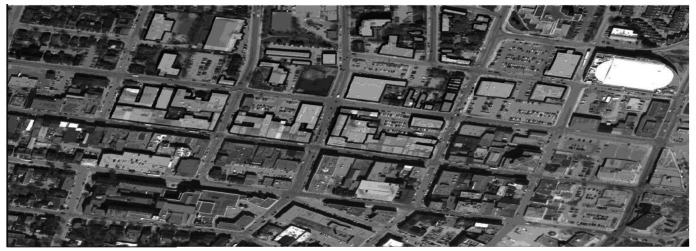
The case study area, a portion of Kingston, Ontario and in particular the Princess Street downtown corridor. The chosen buildings are delineated with bold black lines and greyed over.

A case study was performed in order to develop and test a methodology on a pilot area as seen in Figure 1, which overlaps with the pilot area that was used for a study on PV potential in for Utilities Kingston [27], the local electrical provider for the community. Of 1 km^2^ coverage, the area represents 1% of Utilities Kingston's administrative territory. Data available for the region include tiled DEMs at 0.55 m resolution, tiled aerial photos collected in 2008, tiled LiDAR point clouds acquired in 2008 and a 2010 building roofprint shapefile. LiDAR data acquisition was done in a multi-point discrete return system using an aircraft-mounted Optech Airborne Laser Terrain Mapper 3100. The LiDAR sensor employed a maximum pulse repetition rate of 100 kHz, a scan frequency of 54 Hz and a 20° (half angle) field of view. This resulted in a cross-track resolution of 0.499 m, an along-track resolution of 0.572 m and a swath width of approximately 475 m. The LiDAR data was provided in a raw LAS 1.0 file format and covers the majority of the city of Kingston. For each tile in the database, a raw format .las file, an xyz .txt and a metadata file containing sensor and resolution information were provided. For the tile used for the case study the mean point density is 0.523 m^2^/point.

Among multiple levels of returns that LiDAR offers, the last returns of this area were used for roof construction since they were considered to most likely reach the closest to the ground hence the last object on the ground or the ground itself would be picked up by the last pulses [62,63]. Especially for buildings last and first returns are the same (in z values), which was checked with the current dataset.

**Figure 2.**
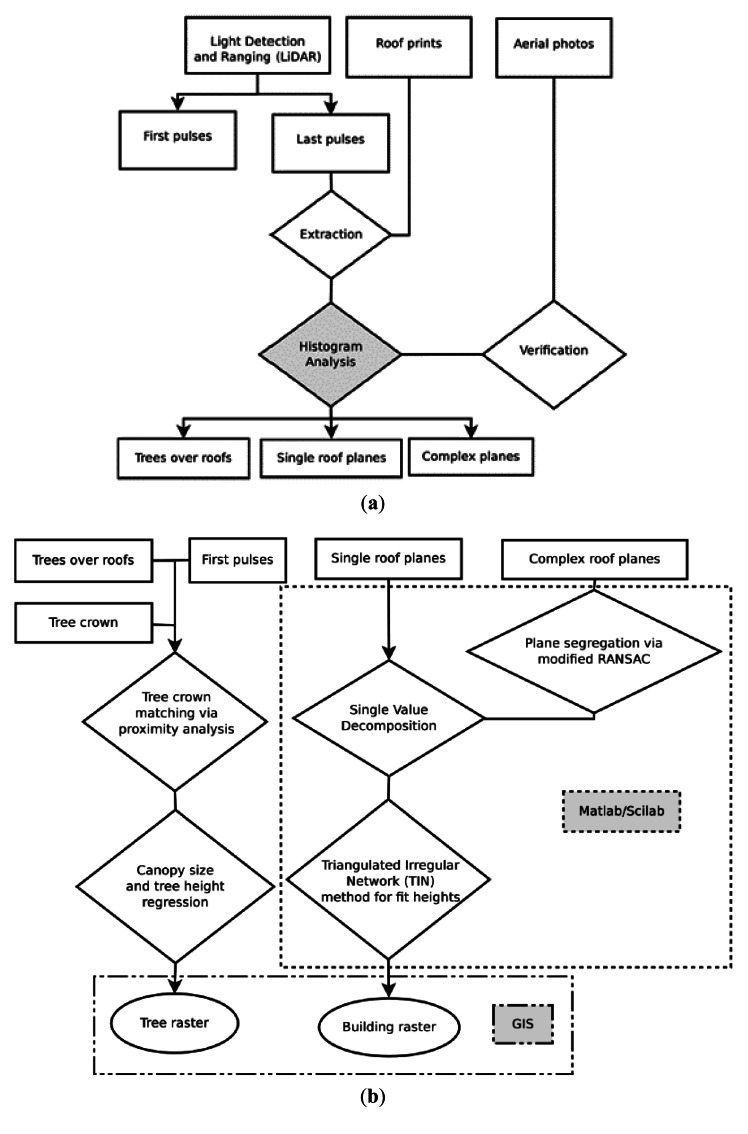

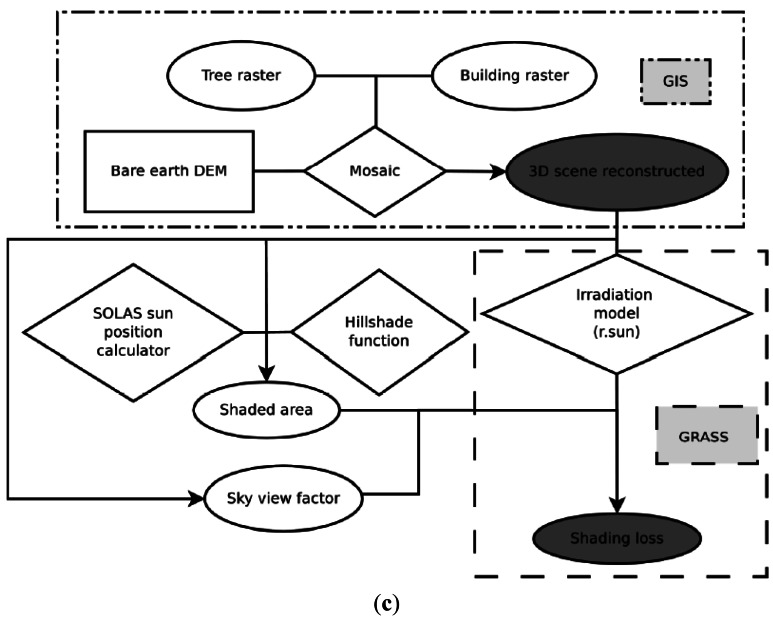
(**a**) Workflow part a. The classification of points based on the objects they correspond to was carried out in ArcGIS; (**b**) Workflow part b. Roof segregation was done using Matlab, followed by the interpolation and rasterization in ArcGIS; (**c**) Workflow part c. Final scene construction (in ArcGIS) and irradiance modeling (in GRASS).

A complete flowchart showing the steps that precede the creation of a DSM presented in this paper and applied for this simulation is given in Figures 2(a–c). In these three figures the triangular envelopes with emboldened border and red label indicate the platform on which the processes and data contained within were carried out. First, the altimetry of last LiDAR return was used for building detection and extraction. The latter was carried out with assistance of the roofprint dataset (an ArcGIS shapefile). The data noise was reduced by using a buffer zone and an elevation floor limit. This is an important process as it allows realistic data to be used for the process. These steps are summarized in Figure 2(a). The remaining “good” data was fit into different planar faces, the procedure of which is explained in Figure 2(b). Figure 2(c) shows how the working geometry is transformed from points to a grid via Triangulated Irregular Network. Accordingly the area grown by TIN (Section 3.4.3) was converted to raster at a resolution of 0.55 m. It was added on top of the existing DEM to create the DSM. It should be pointed out that it is essential that in the mosaic to create a new raster command, the same bit (32 or 16) and data type (floating or continuous) with the DEM is specified. Single points from last returns were extracted from the 3D structures by the Multipart to single part and took 11 minutes. The z values were extracted using the Point to Raster using Shape and took 21 minutes. Next a buffer region around individual roofs was created in order to eliminate the noise occurring along the break lines or edges (Section 3.1). Then an elevation cutoff was used to provide automatic filtering of non-building objects and automatic building detection (Section 3.2). The point cloud was broken into subclouds corresponding to individual buildings, a process in which the buildings points were further refined and trees were concurrently picked out (Section 3.3). After the refinement the subclouds were ready to go into roof fitting (Section 3.4). Computations were carried out on Matlab 9.3 and ArcGIS 9.3 versions on a 4 Gigabytes RAM 64 bites Dell Inspiron Desktop computer.

### 3.1. Buffering the Roof Points

Initial extraction attempts to capture those points that fall within the known outline of a building (from independent data) led to difficulties: since there are independent centimeter to decimeter—level horizontal errors in both the available outlines and the LiDAR points themselves, preliminary capture of “on building” points is imperfect. The LiDAR points within the roofs' outlines still contain incorrectly positioned points representing the ground level height or other objects at slightly lower elevations (e.g., windows on walls, awnings, *etc.*). Lying close to the roof face division lines (breaklines) they hence can disturb the coplanarity of the point cloud, leading to noisy normal vector and hence error in clustering [44,55]. They were eliminated according to their position and height by following the basic procedure described by Haala [60] and Carneiro *et al.* [26], where a building outline is buffered and points in the buffer are considered suspect. Four nominal distances were chosen: ±1 m and −0.5 m. The positive sign means an external buffer; otherwise the buffer lies inside the polygon boundary. Selection by proximity was done first with ground points and then without ground points (ground points are defined to be those below an elevation cutoff—Section 3.2).

### 3.2. Elevation Cut-Off

Next, all points within the roofs' outlines are filtered by introducing a threshold value above the bare earth elevation level (DEM), with the goal being to remove LiDAR points sampled through skylights, into small courtyards, and the like. The same sample was used in determining the appropriate cutoff. Due to the mismatch between when the shapefile, the photos and the LiDAR data were acquired, such a cutoff had to be chosen carefully since any change in building structures in between the time line can make the cutoff appear as capturing redundant or false information such as an abolished patio or a newly built entrance. The largest error was found to be with complexes of roof of different kinds, which were created due to the density of building in downtown Kingston. The roof sample hence was fine-tuned by the removal of these buildings and future work is needed to enable an automated system that can handle complex roof geometries. To determine the range of an appropriate cut off, the zoning bylaw for the township of Kingston was consulted (by-law No. 96–259), which gives the range of 2.5–3.5 m. A vertical cutoff of 2.5 m was the final choice.

**Figure 3.**
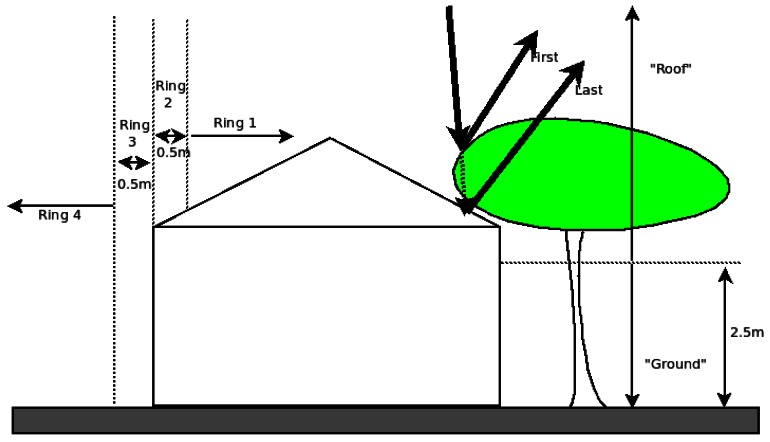
A vertical cut of an example house next to a tree showing the buffer and the elevation cutoff. In addition it shows the vertical extent of the four rings used for classification.

Any features found inside a safe building cloud as determined in Section 3.1 was further filtered to remove points below 2.5 m elevation above the local ground surface. This accounts for points sampled through skylights, small and unmapped courtyards, and the like. Since z values are directly acquired by the LiDAR system, this allows for a higher degree of automatic filtering of non-building objects and automatic building detection as a whole [29] and facilitates precise inner roof segmentation and modeling [44]. Vu *et al.* [64] went as far as using the z values as primary input to delineate the structural information, a translation between a point cloud “world” to a morphological scale space and regarding the behavior of elevation clusters across the scale space as cues for feature extraction. In other words, to Vu *et al.* the definition of an elevation cutoff varies with the object being dealt with [64]. In contrast, in this study, only one elevation cutoff is defined as universal and applied throughout. A vertical slice of the point cloud is included in Figure 3 above, which illustrates the assumptions and definitions for the buffer (Section 3.1) and the elevation cutoff.

### 3.3. Individual Point Subcloud Processing. Tree and Noise Detection

Since the automation algorithms are highly sensitive to any remaining noise, *i.e.*, height jumps, anomaly detection and removal was done [26]: maximum, minimum and average elevations of the points that fall within the roof polygons were used to define an elevation range for each building and hence any elevational anomaly is assumed to belong to signals from rooftop antenna, HVACs, small chimneys, and any overlapping tree canopy. The anomaly detection was checked with corresponding aerial photos and hence the elevational range is building specific. If any point falls out of one standard deviation away from the mean elevation of the point cloud, it is an anomalous signal. Then the areal point cloud was split into individual buildings for further clean up and analysis [54]. As no ArcGIS information for buildings in the city was available, this was done by matching the points to buildings seen on the corresponding aerial photos and by distinguishing the post-processed points by elevation as for most cases, different buildings have different roof structures and heights. Splitting the data into point subclouds also allows registry of buildings as individual entities and electricity users for future assessment of microFIT. On a per building basis, point cloud statistical analysis was carried out to distinguish flat roofs from tilted roofs (Section 4.3). Finally, equations for each roof plane were fit using SVD and mRANSAC.

### 3.4. Roof Fitting

The subpoints at this point are ready to be segmented and used for reconstruction. In a mathematical sense, since the assumption is that each plane can be represented by a distinct equation in the Cartesian coordinates, segmentation is to derive such equations, which are in turns used for interpolation in region growing. Equations for horizontal roof faces are relatively easy to construct, but those for slanted faces have first to be recognized by RANSAC before being linearly regressed by the Singular value Decomposition (SVD) algorithm.

#### 3.4.1. Random Sample Consensus (RANSAC)

A combination of (the concepts of) RANSAC and SVD has been proposed as effective in 3D scene rendering [65,66]: RANSAC is for detecting true outliers and SVD to find the principle components of the roof planes. mRANSAC pseudo-code is more transparent to first time users. Details are provided in previous literature [57–59]. In traditional geometry a plane is a surface that contains straight lines in all directions. On the other hand, mRANSAC considers one plane as the thickness of points ranging between two parallel planes, the distance between which is specified by the programmer. Scripts of RANSAC and SVD available from an online repository (www.mathworks.com) were downloaded and modified such that appropriate considerations were added to the script:

- The number of trials is an interactive input, tuned by different urban topologies and point densities. If the point density is low enough, satisfactory results will be returned after the first run.
- mRANSAC reduces the number of trials with no guarantee for a solution free of gross errors (*i.e.*, obtaining the same result after each iteration [57]). Hence the number of faces is larger than what the actual building does have so that two results for the same face can be compared.
- The number of points of the smallest foreseeable plane surface for each surface becomes one criterion for convergence (the other is the number of trials): larger than necessary of a number of points results in false or under segmentation but smaller than necessary of a number of points will cause the segmented result to have smaller area, compounding the fact that we already used a buffer to select the roof points (Section 3.1).
- Noise points were to be eliminated before running the script (Section 3.3).

#### 3.4.2. Singular value Decomposition (SVD)

The mathematical context of 3D object modeling leading up to the use of SVD is given by Shashua [67] and Sarabandi & Kiremidjian [68]. The algorithm defines a plane as one that best fits the point cloud. Such a problem is a multi-linear optimization in its own right. Both the mathematical proof and the explanation of each command in the script can be found within the vector algebra community. The script was embedded at the end of RANSAC to complete the recognition and regression regarding slanted faces.

#### 3.4.3. Triangulated Irregular Network (TIN)

By this stage, the Z values of LiDAR points had been interpolated according to the equation of the plane to which they are segmented. Since GRASS operates on *ascii* format data these Z values would help turn the shapefile data into grid data via interpolation and rasterization. However they cannot be rasterized right away for risk of losing information [31] or even failure of rasterization when point spacing is too large. Thankfully, the LiDAR points are now ready for plane growing, which is why TIN comes next in the processing chain [56,69]. Besides the disadvantages in the inherent design of the algorithm [56] it also suffers from another difficulty in defining the correct seed surface, which is inevitable for data sets acquired from the urban environments [69]. However after noise removal (Section 3.3 and Section 3.4.1) and homogeneity establishment (Section 3.4.2), the sensitivity to noise is gone and hence the strength of TIN in plane growing comes into full use and helps overcome the dependence on the irregular and sparse nature of LiDAR data [29].

### 3.5. Digital Surface Model (DSM)

The area grown by TIN (Section 3.4.3) was converted to raster at a resolution of 0.55 m, which is the resolution of the provided DEM and approximately the same with the point density of the post-processed point cloud. It was added on top of the existing DEM to create the DSM. The process of mosaicking to create a new raster in ArcGIS requires that the same bit (32 or 16) and data type (floating or continuous) with the DEM is specified. This is the inherent configuration of the command.

## 4. Results

### 4.1. Buffering Size Determination

The effects of each buffer size on the chosen area were investigated by counting the number of points being encompassed by each buffer. Although an inner buffer of 0.5 m has more misclassified points, it actually encompasses more roof points than that of 1 m. A discussion on how using such a buffer will affect the roof area available or solar PV is given in Section 4.3. Here −0.5 m was chosen as a buffer size, or a detection band of 1 m in width.

**Figure 4.**
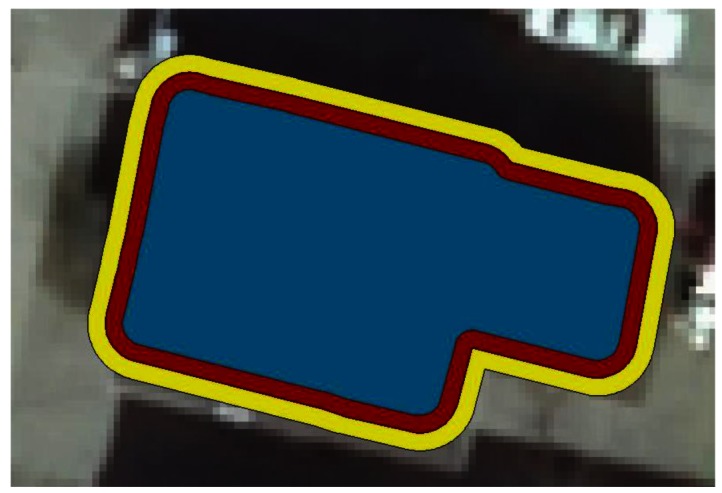
Ring extent: the inner roof area, the outer roof edge, the outer roof buffer and the remaining of the area surrounding a particular building are represented in blue, red, yellow and colorless respectively.

Once it was decided that a suitable buffer size would be 0.5 m, there are four categories of (mis)classification to be populated: (i) ground points that are categorized to be ground (ring 4); (ii) ground points that are categorized to be roof (happening mostly around an outer edge ring of the roof) (ring 3); (iii) roof points that are categorized to be ground (happening mostly in an outer ring of the roof) (ring 2) and (iv) roof points that are categorized to be roof (ring 1). They are represented by blue (ring 1), red (ring 2), yellow (ring 3) and no color (ring 4) in Figure 4 below.

The vertical representation of the rings is given in Figure 3. A sample of 57 completely clean buildings: correct delineation, no trees, simple shape and structure was selected for this error analysis. The test can help identify the source of error. If the cause of error was instrumental (dysfunctional GPS leads to points being misclassified in a wiggly pattern) or geometrical (misclassified points were asymmetrical, either on the outer ring or on the outer edge ring of roofs) then the misclassification matrix will show it. The population can then be used to detect trees and antenna noise. By counting the number of points that spatially fall into each ring the extent of roof is assessed to be correctly classified and captured.

### 4.2. Elevation Cutoff

**Figure 5.**
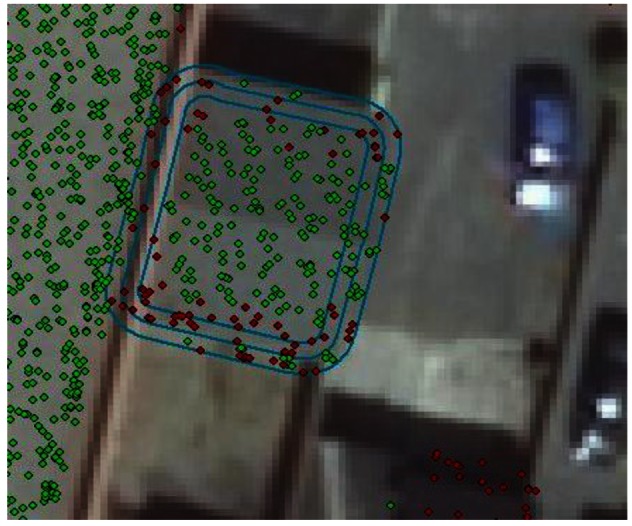
Using 1.5 m as cutoff for small houses helps captures the majority of correct roof points (green) in the roof area and leaves the noise for outer rings.

**Figure 6.**
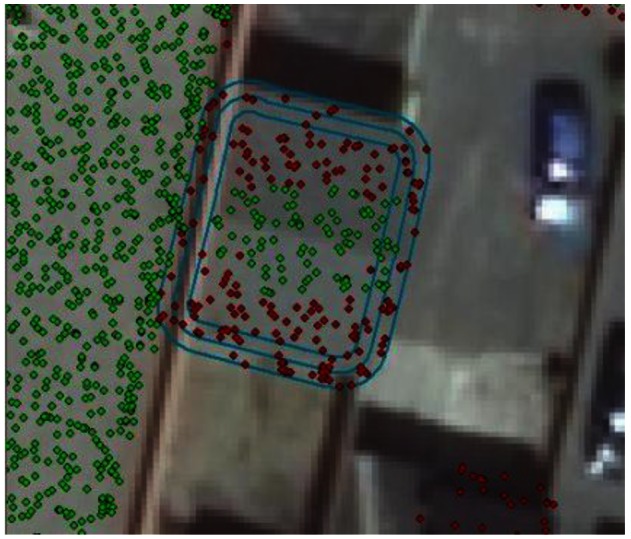
Using 3.5 m as cutoff for small houses eliminates 50% of the correct roof points.

**Figure 7.**
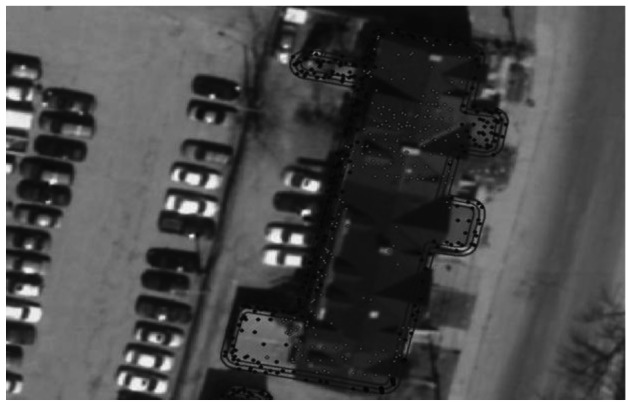
Using 3.5 m as cutoff on large houses points out the discrepancy between the building outline and its aerial photo: the building outline near the upper central part on the photo shows an thin extension formerly belonging to some structure that no longer existed at the time the photo was taken. Using only the out-of-date roofprint would lead to selection of some LiDAR data as legitimate roof.

Based on point counts it was found that (i) 2.5 m or lower is a better cut off for small houses (this should be taken into account when dealing with small houses) and (ii) there are still some left out due to the lack of update for roofprint shapefile, which should be addressed, but may go beyond the ability of the researcher. This is confirmed by observations in Figures 5 and 6, which shows a building with a sloped rooftop, *where green stands for points that meet the cutoff and red otherwise*. On the contrary, 3.5 m is quite effective in picking out bad areas for large houses as can be seen in Figure 7 where an abolished corridor was not updated in the roofprint, but was detected merely by using a higher elevation cutoff. The desired elevation cutoff was thus chosen to be between 3.5 m and 1.5 m.

In order to determine how large the error within the pool of smaller buildings is the following the number of correctly classified points was counted for small houses and large houses within the sample using 187 m^2^ as an area cut off for the first standard deviation and 3.5 m as elevation cut off. However, it should be noted that counting points is a problem because larger entities will have more returns than smaller ones. Although 3.5 m performs better on large buildings it is the small buildings for which care must be taken to prevent under-representation (*i.e.*, losing too much of their roof areas due a stricter cutoff). Hence a 2.5 m cutoff was chosen, which was similar to the value found by Kassner *et al.* [44].

### 4.3. Point Cloud Statistical Analysis

Point cloud statistical analysis was carried out to distinguish flat rooftops from tilted roof planes. The parameters were derived from careful statistical examination of the point clouds and hence are characteristic of only the point spacing and clustering between objects in the explored area. The methodology however can be adapted for other areas yielding characteristic statistics of point spacing and clustering in the area of interest. In general, a single roof plane or roof with minimal differential tilt will have the standard deviation in height among the points between 0 and 0.7 m; flat roofs with obstacles or part of trees will have an elevation standard deviation between 0.7 and 1.4 m; complex roof will have an elevation standard deviation larger than 1.5 m. If the standard deviation is less than 1.4 m, a TIN is checked to see if the roof can be further segmented or a few points can be regarded as trees and exported, or if that is minor noise, the noise will be removed (the noise is obvious when the difference between it and real roof is at least 0.3 m and these points are not close together to form a plane by themselves). The status of a flat roof is confirmed if the histogram is one single spike, or a compound of multiple flat roofs for multiple spikes. For the latter, local maxima will be visited individually and in both cases, the tails of the histograms will represent lower structures (left tail) and roof top obstacles (HVAC, antenna, *etc.*) (right tail). For the case study, 555 point subclouds that cover twice the area chosen as pilot for the Utilities Kingston study were processed in one week, resulting in 1,000 instances of both roofs and trees. The quality of this final point cloud, which is defined in terms of how closely the point cloud matches up with aerial photos and the angles in resultant buildings approach specified blueprint values, is given in the next section.

### 4.4. Error Analysis

**Figure 8.**
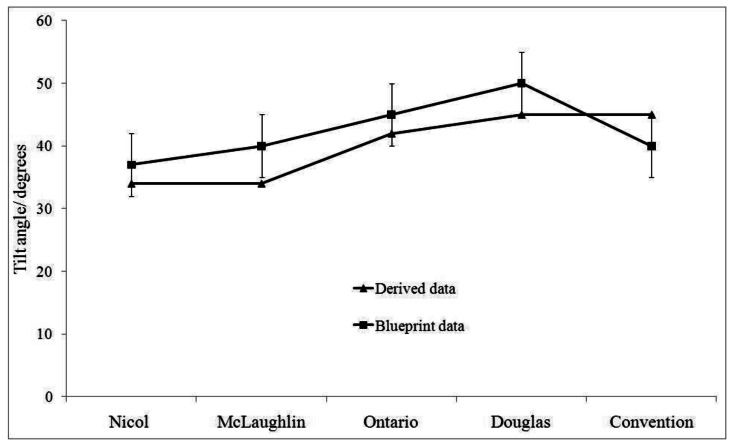
Error analysis of angles for reconstructed selected roofs.

Nyruhuma (2007) assessed how accurate the reconstructed urban scene was to reality using roof angle, roof area and building height [56].van der Sande evaluated the quality of the LiDAR data with reference data [65]. Such valuable ground truth sources for error analysis are not always available and were not accessible for the data set chosen here. However, the performance of the proposed method was evaluated by comparing the roof angles of our results for five complex roof structures (Nicol, McLaughlin, Ontario, Douglas and Convocation Halls) on the Queen's University campus, Kingston, Ontario with those provided in the archived blueprints and shown in Figure 8.

Roof tilt angle, constitutes the surface slope for the DSM, or β, is one of the three defining parameters for the local coordinate system concerning the sun and the site in solar energy engineering [70,71]. It follows that the accuracy of the proposed algorithm, namely of the modified RANSAC and SVD scripts, lies in the precision of the derived roof angles. It was assumed that (i) the roof angle overshadows any plot's terrain, hence once added over the DEM, the roof angle becomes the DSM's local slope; (ii) the number of faces is a user input in the script, which eliminates the need to determine over or under segmentation; (iii) the practice in hand drawing blueprints for the older buildings on campus is to add or subtract 5 degrees from the true angles, which gives rise to a grey zone of 10 degrees around the denoted angular value we have for each face and (iv) elevations checked on the flat roof buildings all returned within 0.20 m of the denoted heights on the blueprints. Given the assumptions, to obtain the angles, the parameters to the equations representing the two planes are computed first. Our SVD script returns the constants a, b, c, d of the plane equation:

(1) a∗x+b∗y+c∗z=d

In particular, the normal vector to this plane can be written in row form as [a, b, c]. The angle between two planes:

(2) $$a_{1}*x_{1}+b_{1}*y_{1}+c_{1}*z_{1}=d_{1}$$

and:

(3) $$a_{2}*x_{2}+b_{2}*y_{2}+c_{2}*z_{2}=d_{2}$$

The corresponding normal vectors are (a_1_, b_1_, c_1_) and (a_2_, b_2_, c_2_), is given by the inverse cosine of the dot product between two normal vectors:

(4) $$\theta ={\text{cos}}^{-2}\left[\frac{\left(a_{1}*a_{2}+b_{1}*b_{2}+c_{1}*c_{2}\right)}{\sqrt{\left(a_{1}^{2}+b_{1}^{2}+c_{1}^{2}\right)}*\sqrt{\left(a_{2}^{2}+b_{2}^{2}+c_{2}^{2}\right)}}\right]$$

There were only five buildings with available blueprints, and both tilted roofs and good LiDAR records. As can be seen in Figure 8 the LiDAR-derived angles fall into the error zone of ±5 degrees from the blueprint values. A specific case (McLaughlin Hall) has a series of dormers along its front and back roofs that resulted in exceptionally poor LiDAR point cloud quality as the sensor picked up part of the dormers and part of the roof in between the overhangs.

## 5. Discussion and Future Work

As can be seen in Figure 8, the results are within 5% error given five key assumptions: (i) individual buildings can be modeled properly by a composition of planer faces; (ii) anything below a chosen elevation cutoff is irrelevant for solar PV potential assessment; (iii) tree canopies are opaque; (iv) small windows on the roofs, overhangs on the walls, HVACs and antennas do not occupy so large a space that their omission adds significant area to the roof area free for PV panels; (v) the height of the object and subsequently the altimetry of the DSM is the difference between LiDAR's Z values and the DEM; and (vi) no discrepancy in real time urban structures between aerial photos (AP) and LiDAR. For Kingston, which lies on the latitude of 44.6 a discrepancy of tilt angle of 5 degrees from optimal will reduce the PV yield by 1 to 5% as determined by PVSyst.

Neither will it affect the validity of the DSM as the elevation is preserved. The roof area, however, will be smaller, since the roof area was twice reduced: the first time by the use of a buffer along the edges and the second time by the point thinning effective in the RANSAC script. That means the output would be a conservative estimation of area available for PV panels. In actuality, a rooftop PV system often takes up between 40 and 80% of the roof area [26].

The methodology presented here is a continuation of previous attempts made by Kassner *et al.* [40], Dorninger & Pfeifer [25], Jochem *et al.* [31] and Carneiro *et al.* [26] to provide urban rooftop data for determination of the regional PV potential on rooftops. However, this method is more interdisciplinarily transparent, comprehensive and has the advantage of relaxing the mandatory data quality. On its own it is the next piece of the pyramidal procedure to estimate solar photovoltaic potential from a regional level [23], to a municipal level [22,27,72] and now a household scale. Given these qualities, it is suitable for use in regions without good LiDAR data and part of it is possible for utilities in the developing countries where these techniques are at an early stage of development. The weakness of this methodology is a compromise between mathematical sophistication and technical adaptability, between automation and intensive supervision. For example, the role played by z values in building extraction is less flexible than that in Vu *et al.* [64]. Although the results were confirmed to be in good agreement with blueprints it should be recognized that in a different urban setting, e.g., India, Iran or Southeast Asia, the styles and scales of architecture will be more varied. Such complex architecture calls for a more relaxed scheme of elevation cut-off, but also requires a large scale comparative study of urban morphology in order to develop such scheme. Technicality forms only part of the methodology design. The choice of different components was reached at the convergence of time, personnel, data (quality) and available expertise.

In light of a variety of users (utilities planners and project developers, municipal governments, individuals with ArcGIS skills and interest in renewable energy, and renewable energy advocates) and their needs, an attempt was made here to give a comprehensive examination of options and suggestions for future work. The aspects mentioned in this paper, mainly pertaining to the integration of spatial information in energy modeling, are among the bottlenecks to the process of integrating solar PV (and other forms of renewable energy) into the current electricity grid. The final piece of the pyramidal process is to run shading and irradiation simulation on a micro-site, namely a sample size of about 30–50 households in downtown Kingston [27,72]. Since there are not many trees, let alone tall trees, in downtown Kingston, the tree handling is left for future work. While extracting buildings it was also possible to extract trees. The approximation of tree canopies is left as a separate puzzle, which is very much related to that of curved surface. It so happened that the chosen (micro) site has no curved surface, which otherwise can be approximated by as a set of planar faces [25].

The acquired experience in running the SVD script can be incorporated into future works to improve their performance. Since the algorithm itself is random, not all the same points and only slightly different planes will be output anytime a run is attempted on a particular roof structure or roof facade. To facilitate the completion of the process the following parameters need to be fine-tuned: (i) specify more than the actual number of roof faces, lest the program terminates before all the roofs are recognized; (ii) only use 80% of the estimated number of points corresponding to one plane to segment out the plane in question; (iii) use the number of points representing the most dominant face in the structure *first*, hence the remaining point cloud is even easier to resolve and (iv) in some cases of bad data quality, the raw z values pertain the true roof angles, but the interpolated ones do not. Hence it is suggested to include in the homogeneity criteria the minimization of the angular discrepancy between the raw and the interpolated z values.

This study extended the argument made by Sunak & Madener [39] and Manfren *et al.* [73] on the shifting paradigm and confirmed the vision by such authors as Buchanan & Brussel [74] and Brenner [24]: remote sensing will play an important role not just in the paradigm shift of urban energy, but in urban planning as a whole. It will facilitate the prediction and management of urban sprawl [75–77] and push forward sustainable urban planning in not just the developed world, but also the developing countries as they catch on with the technologies [46,49]. The technical and hence the perceived barriers to similar topics of research are taken down thanks to the facts that the data here is the “worst” case scenario that one can have for a similar project and that the workflow was developed for a diverse community ranging from engineers, spatial analyst to urban and social planners. To that end, open sourcing the methodology and software is another pressing topic, which could help further accelerate the paradigm shift.

## 6. Conclusions

The paper provides a methodology for the application of LiDAR to automated solar photovoltaic deployment analysis on the regional scale. Challenges in urban information extraction and management for solar PV deployment assessment are determined and quantified. First, a comprehensive examination and comparisons of existing algorithms and approaches to turn LiDAR point cloud into 2.5D urban scenes was provided. A more cross-disciplinarily transparent methodology that attains a 95% accurate segmentation from raw and randomly chosen data was demonstrated. The methodology implements what previous literature recommends in terms of integrating cross disciplinary competences in remote sensing, GIS, computer vision and urban environmental studies. It is a robust methodology that can work with poor-quality data and reconstruct vegetation and building separately but concurrently. Since the coarse selection of building regions is crucial to reliable results considerable attention was focused on this first step. Subsequent steps in building extraction, segmentation and reconstruction were carried out accompanied with mathematical proofs and illustrations. The approach was data driven hence the whole attempt can be regarded as a large scale optimization problem aiming at best approximating the point cloud. Singular Value Decomposition, Random Sample Consensus and Triangular Irregular Network were confirmed as essential tools for the task. Rules of thumb were collected to incorporate in the development of such scripts for extracting rooftops for solar photovoltaic potential. But there is still room for the more mathematically rigorous or biologically minded audience to contribute and orient the workflow to suit their needs. Hence this can be regarded as the next step towards a new generation of urban analysis software.
